# Amphistome Infection and Species Diversity of Freshwater Snails Collected from Selected Wildlife Drinking Water Sources in Matebeleland Region of Zimbabwe

**DOI:** 10.3390/vetsci11050211

**Published:** 2024-05-13

**Authors:** Madeline Siyazisiwe Sibula, Mokgadi Pulane Malatji, Cosmas Nyahunda, Samson Mukaratirwa

**Affiliations:** 1 School of Life Sciences, College of Agriculture, Engineering and Science, University of KwaZulu-Natal, Westville Campus, Durban 4001, South Africamukaratirwa@ukzn.ac.za (S.M.); 2 Department of Applied Biology and Biochemistry, Faculty of Applied Science, National University of Science and Technology, Bulawayo P.O. Box AC939, Zimbabwe; 3 One Health Centre for Zoonoses and Tropical Veterinary Medicine, Ross University School of Veterinary Medicine, Basseterre P.O. Box 334, Saint Kitts and Nevis

**Keywords:** gastropods, identification, diversity, amphistomes, molecular detection, Zimbabwe

## Abstract

**Simple Summary:**

Amphistomes, also known as rumen flukes, are parasites of domestic and wild ruminants and occur globally. Adult parasites are found in the forestomach, and the young flukes are found in the small intestines, causing severe damage. The flukes are transmitted by various species of freshwater snails. While the disease is well documented in domestic ruminants, there are gaps in knowledge pertaining to wild ruminants with regard to the fluke species as well as the snail species which transmit them. Therefore, freshwater snails were surveyed from 19 water points that are frequented by wild ruminants in the Matebeleland region, Zimbabwe. Snails were found at nine sites, and eight species were identified and screened for rumen fluke DNA to determine the fluke species and prevalence of infection. Rumen fluke DNA was detected in 11.9% of the screened snails. Prevalence was high in the West Nicholson locality and in *Bulinus globosus* snail species. One rumen fluke species, i.e., *Calicophoron microbothrium*, was confirmed in one snail species and there were also mixed infections with lung fluke parasite, *Paragonimus* spp., in two snail species. This was the first study documenting the presence of this lung fluke in Zimbabwe.

**Abstract:**

This study aimed at determining the identity of freshwater snails collected from selected water habitats frequented by wildlife as source of drinking water in the Matebeleland region of Zimbabwe and further screening the identified snails for natural infections with amphistomes using PCR. A total of 487 freshwater snails were collected from six areas in the Matebeleland region of Zimbabwe for identification and screening of amphistome infection. Eight freshwater snail species were morphologically identified and *Biomphalaria pfeifferi, Bul. tropicus, Bul. truncatus, Bul. globosus*, and *L. (R.) natalensis* were confirmed using the COI gene. *Bulinus tropicus* and *Phy. acuta* were the most abundant species at 33.9% (165/487) and 31.2% (155/487), respectively. DNA of amphistome was detected in 11.9% (58/487) of the collected snails. The highest infection rate was detected in *Bul. globosus* (44.4%). West Nicholson recorded the highest infection rate (33.9%), and infection was not detected in *L. (R.) natalensis*, *Phy. acuta*, and *Bellamya* spp. Amphistome DNA from *M. tuberculata* was successfully sequenced and identified as *Calicophoron microbothrium*. An additional band was detected in *M. tuberculata*, *Bul. tropicus*, and *Bul. trancatus*, which showed a 96.42% similarity to *Paragonimus* sp. sequence in the GenBank.

## 1. Introduction

Amphistomes are digenetic trematodes that exhibit a heteroxenous life cycle that includes an intermediate and definitive host [1]. Although over 70 amphistomes have been recorded globally to date [2,3], the intermediate host snails have only been described for a few amphistomes species in sub-Saharan Africa [4]. Amphistome species use several freshwater snail species as intermediate hosts (IHs) for transmission, and these include species from the genus *Bulinus* [5,6,7], *Galba* [8,9], *Lymnaea* (*Radix*) [10,11], *Biomphalaria* [12], and *Segmentorbis* [12]. According to Laidemitt et al. [12], *Bulinus* species, which are the most widely distributed snails in sub-Saharan Africa, accounts for the wide distribution of *Calicophoron* spp. in Africa. Furthermore, several *Bulinus* species have been implicated in the transmission of *Calicophoron microbothrium*, an amphistome responsible for most cases of amphistomosis in both wild and domestic ruminants [4].

Like other trematodes, both domestic and wild ruminants get infected with amphistomes by grazing in pasture around drinking water points contaminated with metacercariae [13]. Infection in wild ruminants can be confirmed at post-mortem by the presence of immature stages in the duodenum, and adult amphistomes in the forestomachs of ruminants, and at ante-mortem through the detection of eggs in feces [14]. However, surveys and monitoring of these infections in free-ranging wild ruminants can be challenging [15] due to factors such as accessibility and ethical issues associated with capturing, handling, and releasing wild animals to collect non-invasive fecal samples [16]. Moreover, identification up to the species level using morphological characters is a challenge, and at ante-mortem, the egg morphology is not easily distinguishable from that of other trematodes such as *Fasciola* spp. [17].

Data from previous research in sub-Saharan Africa have indicated that majority of amphistomes species occurring in wild ruminants are also common in domestic ruminants, except *Bilatorchis papillogenitalis*, *Carmyerius bubalis*, and *Cotylophoron macrosphinctris*, which have only been documented in wild ruminants to date [4,18]. These three species are amongst those with unknown intermediate snail hosts. Amphistome infection in the intermediate snail hosts can be detected through shedding of cercariae or squashing of the snail soft tissue and visualizing the developmental stages using a light microscope and the subsequent morphological characterization of the cercariae/rediae/sporocyst [19,20,21]. However, the morphological characterization of these larval stages has limitations as most studies could only discriminate up to the genus level [21,22,23], with only a few studies able to distinguish up to the species level [24,25,26,27]. Nonetheless, Kane et al. [28] indicated that morphological identification of larval trematodes is error-prone and hard or impossible for the genus or species level resolution.

Although some recent studies still utilize shedding of cercariae as sole detection method of infection [21], most recent studies couple the use of shedding cercariae with molecular techniques such as Restriction Fragment Length Polymorphism (RFLP) of Polymerase Chain Reaction (PCR) products [29] or sequencing of the PCR products for identification up to the species level [6,12,29]. Some studies also amplified the larval DNA directly from the snails, followed by sequencing [12,30] or RFLP of the products [29]. However, Schols [30] showed that detection of larval infection using PCR was better than cercariae shedding.

According to Carolus et al. [31], knowledge on the prevalence, diversity, and ecology of both intermediate host freshwater snails and trematodes is key to understanding disease transmission dynamics and the possible control of trematodes of economic significance. Although there are several ways of identifying freshwater snails and trematode larval stages based on morphological characters, molecular methods are known to provide better resolution for identifying snails and trematodes at the species level [32]. Hence, the aim of this study was to determine the identity of freshwater snails collected from selected water habitats serving as water sources for wildlife in selected game ranches, conservancies, and game parks located in the Matebeleland region of Zimbabwe and the amphistome species they may transmit in these habitats.

## 2. Materials and Methods

### 2.1. Study Areas and Sample Collection

**Figure 1:**
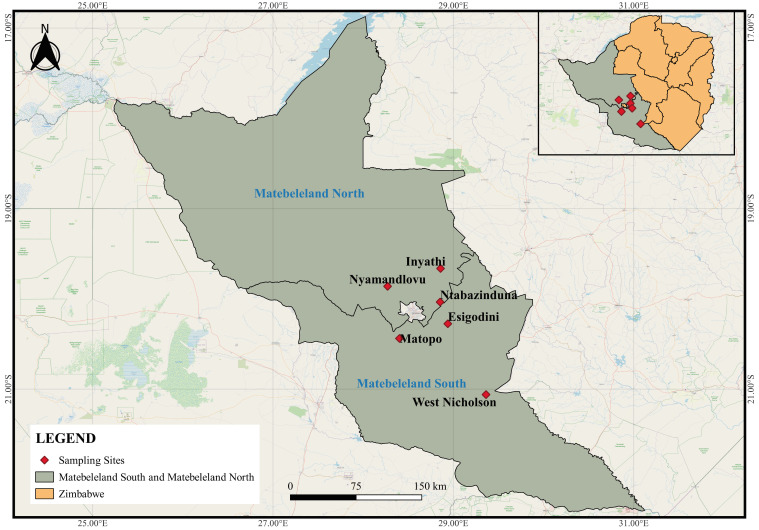
All sampling sites of the study located in the Matebeleland region of Zimbabwe.

Freshwater gastropods were surveyed from water sources frequented by wildlife around game ranches, conservancies, and game parks located in Inyathi, Nyamandlovu, and Ntabazinduna, located in Matebeleland North and Esigodini, West Nicholson, and Matopos which are in Matebeleland South province of Zimbabwe (Figure 1). Sampling sites were pre-selected based on the following criteria: little or no human activity and locality is a drinking water point for wild ruminants. The habitat type and vegetation cover were recorded for each site (Table 1), and snails were collected using a scooping net and dredge for superficial and deep-water samples, respectively [31]. Collected snails were first washed with distilled water and thereafter preserved in 70% ethanol for morphological and molecular analyses.

### 2.2. Morphological and Molecular Identification of Snails

#### 2.2.1. Morphological Classification

Morphological identification of snails was based on the classification keys as described by Brown [33]. Snails of the same species morphologically were grouped, and representative specimens were selected for confirmation using PCR.

#### 2.2.2. DNA Extraction from Gastropods

Selected snail specimens were washed with sterile water to remove alcohol, and excess water was removed with sterile absorbent paper. Tissue from foot of each snail or whole snail tissue was harvested using either a sterile blade or the whole snail tissue was removed and then frozen at −20 °C for 2 h before use. DNA was extracted using a modified Quick-DNATM Tissue Miniprep Kit (Zymo Research Corporation, Irvine, CA 92164, USA) protocol.

#### 2.2.3. Molecular Characterization of Gastropods

Extracted DNA were amplified based on the COI region using the primers COI (F) 5′-TAATGTWATTGTTACAGCACATG-3′ and COI (R) 5′-GTTGRTATAAAATAGCATCACCW-3′ [31]. PCR was performed in a total reaction volume of 25 µL, composing of 5 µL of 5X One Taq PCR buffer, 0.4 µL of 10 mM dNTPs, 0.5 µL forward primer (10 mM), 0.5 µL reverse primer (10 mM), 3 µL of 25 mM MgCl_2_, 2 µL DNA template, 0.125 µL of One Taq polymerase (NEB, Hitchin, UK), and nuclease-free water to make up the final volume. The cycling protocol for the reaction was as follows: initial denaturation at 94 °C for 3 min, followed by 30 cycles of denaturation at 94 °C for 30 s, annealing at 64 °C for 45 s, extension at 68 °C for 1 min, and a final extension of 68 °C for 15 min. The fragments were separated on 1% agarose stained with ethidium bromide, and successful amplicons were identified by a band at 630 base pairs. Amplicons were sent to Inqaba Biotech for Sanger sequencing.

#### 2.2.4. Molecular Detection of Amphistomes from Snail Tissue Samples

Snails were assessed for amphistome infection using the primers GA1 (5′-AGAACATCGACATCTTGAAC-3′) and BD2 (5′-TATGCTTAAATTCAGCGGGT-3′) [12]. *Calicophoron microbothrium* DNA was used as a positive control/signal for amphistome DNA. The PCR mix was composed of 2 µL of 10X PCR buffer SuperTherm (Separation Scientific SA (Pty) Ltd., Roodepoort, South Africa), 0.8 µL of 10 mM dNTPs, 0.4 µL forward primer (10 µM/µL), 0.4 µL reverse primer (10 µM/µL), 1.6 µL 25 mM MgCl_2_, 1 µL DNA template, 0.2 µL Super Therm polymerase, and nuclease-free water to make up a total reaction volume of 20 µL. Amplification was performed under the following thermocycling conditions: 95 °C for 3 min, followed by 30 cycles of 95 °C for 30 s, 55 °C for 45 s, and 72 °C for 1 min, and a final extension of 72 °C for 15 min. Amplicons were separated on 1% agarose gels stained with ethidium bromide, and positive isolates were identified by a band at approximately 385 bp. Positive amplicons were sent to Inqaba Biotechnical Industries (Pretoria, South Africa) for Sanger sequencing.

#### 2.2.5. Molecular Analysis of Trematode/Amphistome Isolates from Snails

Sequences were viewed, assembled, and manually edited using BioEdit version 7.2 (Sequence Alignment Editor) [34], and NCBI BLAST (Basic local alignment search tool) was used to identify the closest matches available on GenBank database. Sequences were trimmed to a common length of 455 nucleotides. Tamura 3-parameter (T92) was selected as the best model fit for the dataset, and the neighbour-joining (NJ) and maximum likelihood trees were generated on the MEGA 7 software [35]. The phylograms were 50% majority-rule, and the nodal support was estimated using 1000 bootstrap pseudo-replicates for both methods.

## 3. Results

### 3.1. Description of Snail Habitats

From the six localities visited in the Matebeleland region (Figure 1), 19 water sites were surveyed, and these were predominantly man-made waterholes in Nyamandlovu (n = 13), dams in West Nicholson (n = 2), Matopos National Park (n = 1), Ntabazinduna (n = 1), and Esigodini (n = 1), and lastly, a river in Inyathi (n = 1) (Table 1). All habitats had different types and levels of submerged vegetations, and only habitats from West Nicholson, Esigodini, and Inyathi had trees on their periphery. The surrounding areas were clear, with decaying matter found in dams in West Nicholson and Ntabazinduna. Results show that although all 13 sites from Nyamandlovu had solely wildlife activity, some sites showed a level of interaction or shared habitat with livestock (n = 4) and to a lesser extent humans (n = 1).

### 3.2. Morphologically Identified Snail Species and Their Abundance

Snails were found at 9 of 19 sites (47.37%), while none were found at 10 sites from Nyamandlovu (Table 1). A total of 487 gastropods were collected, and these were from Nyamandlovu (n = 212) and Ntabazinduna (n = 174), followed by West Nicholson (n = 59), Esigodini (n = 21), Matopos National Park (n = 18), and to a lesser extent Inyathi (n = 3) (Table 2). From these collections, eight species were morphologically identified as *Melanoides* (*M*.) *tuberculata*, *Bulinus* (*Bul*.) *globosus*, *Bul*. *truncatus*, *Bul*. *tropicus*, *Biomphalaria* (*Bio*.) *pfeifferi*, *Physa* (*Phy*.) *acuta*, *Lymnaea* (*L.*) *natalensis*, and *Bellamya* spp. (Table 2).

*Bulinus tropicus* was the most distributed species across sites and was found in four of the six surveyed areas. Furthermore, this species was the most abundant and contributed 33.9% (165/487) of the collected snail populations. This was followed by *Phy. acuta* that contributed to 31.2% (155/487) and was found in Esigodini and Nyamandlovu. *Bulinus truncatus* and *M. tuberculata*, which were each found in Ntabazinduna and West Nicholson, contributed to 12.5% (61/487) and 11.9% (58/487), respectively. Surprisingly, *Bio. pfeifferi*, *Bul. globosus*, and *L. natalensis* were found in multiple areas, though in low numbers of 20/487 (4.1%), 18/487 (3.7%), and 8/487(1.6%), respectively. The least collected snail species was *Bellamya* spp., which was found in Ntabazinduna and contributed to 0.4% (2/487) of the collected snail population.

### 3.3. Molecular Confirmation and Phylogenetic Relationship of Snail Species.

**Figure 2:**
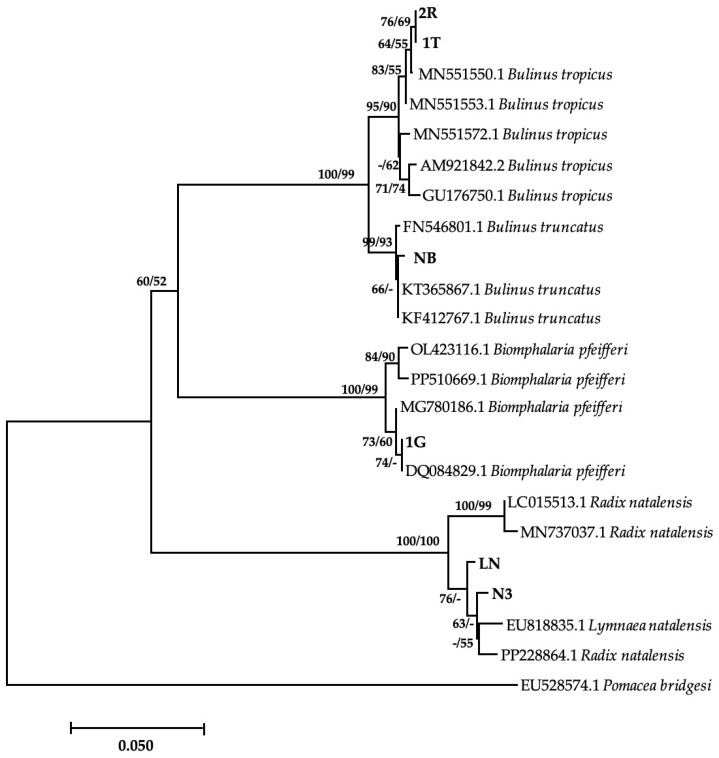
Neighbor-joining tree-based COI gene illustrating the relationship between freshwater snails obtained from game ranches, conservancies, and game parks located in the Matebeleland region of Zimbabwe and the closest matches from the NCBI GenBank. The nodal support values (%) indicated in an order using neighbor-joining and maximum likelihood. 2R, IT, NB, 1G, LN, N3 = isolates from the study. The 50% majority-rule was applied, and any support value of lower than 50% was represented by a hyphen.

Of the eight morphologically identified species, four snail species were confirmed with a BLAST similarity index ranging between 98.24 and 100% (Table 2). Amongst these was 1G, which showed 100% homology with *Bio. pfeifferi* from Zimbabwe (DQ084829). However, the phylogenetic tree showed a moderate support between this isolate and other GenBank isolates (Figure 2). The isolated *Bio. pfeifferi* formed a well-supported sister clade to *Bulinus* species, which falls under the same family. Our isolate NB showed a 99.78% identity with *Bul. truncatus* isolate from Iran (KT365867) and formed a well-supported clade with other *Bul. truncatus* isolates. These isolates formed a strongly supported monophyletic sister clade with *Bul. tropicus* isolates, including those from this study (2R and IT) (Figure 2), which showed 99.78% homology with *Bul. tropicus* from Uganda (MN551550). Lastly, our *L. natalensis* isolates (LN and N3) (Figure 2) showed a homology of 98.68% with *L. natalensis* from Malawi (EU818835). These species formed a well-supported separate clade from the *Bulinus* and *Biomphalaria* clade. However, the relationship between the isolates was moderately to weakly supported. The sequences generated from this study were submitted to NCBI GenBank under the accession numbers PP389543–PP389548. Sequences for *Bul. globosus* failed quality control, while sequencing failed for *M. tuberculata*, *Phy. acuta*, and *Bellamya* spp. The time lapse between the PCR and the amplicons reaching South Africa from Zimbabwe for post clean-up and sequencing might have compromised the quality of the PCR products, and possibly causing their degradation.

### 3.4. Molecular Detection of Amphistome DNA in Field-Collected Snails

All 487 gastropod isolates were individually screened for amphistome DNA based on the ITS-*2* gene. A total of 58 of 487 (11.9%) snails showed a band at approximately 385 bp, which was consistent with a *Calicophoron (Cal.) microbothrium* isolate used as a control for amphistomes. *Bulinus globosus* recorded the highest prevalence of amphistome infections (44.4%), followed by *M. tuberculata* (33.9%) (Table 3). No amphistome DNA was detected in *Phy. acuta*, *L. natalensis*, and *Bellamya* spp. The highest incidence of amphistome infections in snails per locality was recorded in West Nicholson, with a prevalence of 33.9% in *M. tuberculata*. In contrast, snails from Inyathi did not harbor any amphistomes, and those from Nyamandlovu had a prevalence of 2.4% in *Bul. tropicus* despite the area recording the highest number of gastropod specimens collected. However, sequencing of amphistome amplicons was only successful for a sample from *M. tuberculata*, which was confirmed as *Cal. microbothrium* with a percentage similarity of 100% (Table 4) and submitted to GenBank under the accession number of PP392962. The remaining samples, which were the majority, failed quality control.

**Figure 3:**
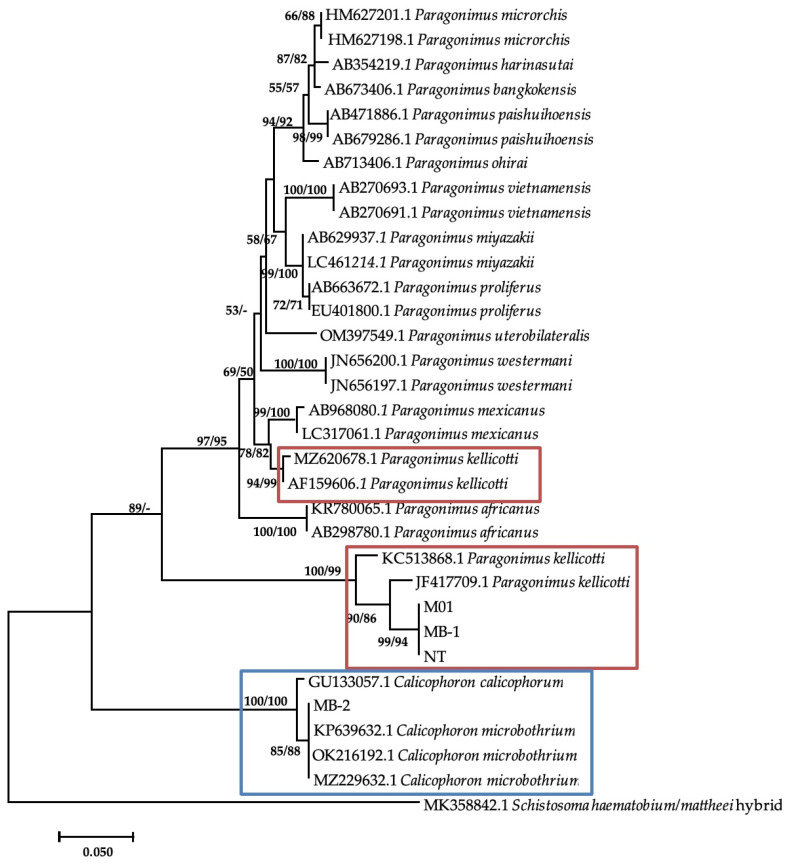
Neighbor-joining tree-based ITS-2 gene illustrating the relationship between trematodes isolates from freshwater snails from the Matebeleland in Zimbabwe and the closest matches from the NCBI GenBank. The nodal support values (%) indicated in an order using neighbor-joining and maximum likelihood.

Additional bands were observed at approximately 280–290 base pairs from *M. tuberculata* (n = 9) and *Bul. tropicus* (n = 1), from Esigodini and *Bul. truncatus* (n = 1), and from Ntabazinduna, indicating a mixed infection. Sequencing of three of the additional bands from *M. tuberculata* and BLAST analysis showed a similarity index of 96.42% to *Paragonimus kellicotti* (Table 4). The sequences were submitted to GenBank as *Paragonimus* sp. under the accession numbers of PP392960, PP392961, and PP392963. Phylogenetic analysis showed that these sequences formed a clade with two *P. kellicotti* isolates from India (KC523868.1) and the United States (JF4177709.1) from *Indoplarnobis exustus* and *Orconectes virilis*, respectively. The genetic distance within this clade was 3%. This clade also formed a monophyletic sister clade with various *Paragonimus* species, including two more *P. kellicotti* (Figure 3). The genetic p-distance between these two clades was 28%.

## 4. Discussion

Snail survey conducted in this study was from three different habitat types that include dams, waterholes, and a river. All the habitats had macrophytes (submerged vegetation), with Ntabazinduna sampling site having an abundance of brown algae macrophytes. According to Min et al. [36], physico-chemical properties and macrophyte abundance together with other factors influence the diversity of freshwater snails. The most abundant snail species were collected from Ntabazinduna, accounting for 36% of the total number of snails. Middelboe and Markager [37] suggested that the presence of submerged macrophytes increase periphyton, the food source of *Bulinus* spp. and *Bio. pfeifferi*. In this study, a total of eight snail species collected from the Matebeleland region of Zimbabwe in selected areas included *Bul. globosus*, *Bul. tropicus*, *Bul. truncatus*, *M. tuberculata*, *Bio. pfeifferi*, *L. natalensis*, and *Phy. Acuta*, and these have been implicated in the transmission of amphistome species in wildlife and domestic ruminants [4] except *Phy. acuta* and *Bellamya* spp.

Results showed that *Bul. tropicus* was the most widely distributed and abundant species in the Matebeleland region of Zimbabwe. This was not surprising as earlier reports have indicated that this species is the most widely distributed freshwater snail in Zimbabwe [38] and is well adapted to a wide variety of environments [33]. However, Chingwena et al. [7] later reported that *Bul. tropicus* was the most abundant species in the lowveld and the third most abundant snail species following *L. natalensis* and *Bul. globosus* in the highveld. Although this still explains the abundance of this species in the Matebeleland region that falls within the middle to highveld, *L. natalensis* and *Bul. Globosus* were surprisingly collected in low numbers in this study. *Physa acuta*, the second most abundant species, is an invasive snail, which was previously reported in Zimbabwe [39]. Although this species showed a limitation in distribution, and the one locality where this snail species was collected had macrophytes in abundance compared to other species. This is not surprising as not only several authors have shown that this species is now widely distributed in Zimbabwe [40] but also that it is invasive [41].

Of the eight snail species screened for amphistome DNA, *M. tuberculata*, *Bio. pfeifferi*, *Bul. tropicus*, *Bul. globosus*, and *Bul. truncatus* were positive. Amphistome detection in the *Bulinus* and *Biomphalaria* species was not surprising as a wide range of these snail species have been reported to act as intermediate hosts for different amphistome species in sub-Saharan Africa [7,25,42,43,44]. Although *Bul. tropicus* was reported to act as an intermediate host of *Cal. microbothrium*, with high prevalence of experimental infections [7], our study reported a high prevalence of amphistome DNA in *Bul. globosus*. *Melanoides tuberculata* showed the second highest prevalence of amphistome infection in this study. Previous reports have already confirmed the susceptibility of this species through experimental infections with *Cal. microbothrium* in Zimbabwe [7] and South Africa [2], and to another trematode species, *Gastrodiscus aegyptiacus*, in Zimbabwe [45]. Based on the successful identification of *Cal. microbothrium* from *M. tuberculata* through sequencing, to the best of our knowledge, this is the first study to confirm natural infection of this Thiaridae species with *Cal. microbothrium* in Africa.

Results showed that amphistome DNA was not detected in three snail species, namely, *Phy. acuta*, *Bellamya* spp., and *L. natalensis*. Although earlier experimental studies have showed that *L. natalensis* was refractory to *Cal. microbothrium* [7], this species was found infected with *Gastrothylax*/*Paramphistomum* in Zimbabwe [30]. A similar pattern was observed with *Phy. acuta*, which was successfully infected with *G. aegyptiacus* during dissection after experimental exposure [45], but no amphistome DNA was detected from this species despite being collected in high numbers, indicating that there might be amphistome species IH specificity, and this may be confirmed through experimental studies like those conducted by Chingwena et al. [7].

Results from this study revealed cases of mixed infections of amphistome DNA with other DNA of a trematode species identified as *Paragonimus*-like species in *Bul. truncatus* and *M. tuberculata*. *Paragonimus* spp. are lung flukes that have been reported worldwide including Africa and mainly in West and Central African regions. Species reported in the West and Central Africa include *P. africanus*, *P. gondwanensis*, *P. kerberti*, and *P. uterobilateralis* [46]. According to Procop [47], *Paragonimus* species usually utilize snail species from families Pleuroceridae and Thiaridae as IHs. While results from this study and previous reports link paragonimiasis with presence of *M. tuberculata* in South Africa [48], which may explain the infections observed in *M. tuberculata* in this study, the phylogenetic positioning of these isolates and the high genetic distance with other reported *Paragonimus spp.* raise concerns of whether these isolates belong to the genus *Paragonimus*, or the sequences deposited on GenBank are misidentified under the genus *Paragonimus*.

## 5. Conclusions

Eight freshwater snail species common in drinking water sources frequented by wildlife from the Matebeleland region of Zimbabwe were identified in this study with *Bul. tropicus* and *Phy. acuta* as the predominant species, and the overall prevalence of amphistome DNA (*Cal. microbothrium*) was 11.9%. Prevalence rate was higher in *Bul. globosus* followed by *M. tuberculata*. The highest number of snails were collected from Nyamandlovu, meanwhile the highest prevalence of amphistome DNA was recorded in Beitbridge. A single sample was confirmed as *Cal. microbothrium* through DNA sequencing and a mixed infection with *Paragonimus* species-like trematode was also confirmed. Failure to sequence other amphistome samples shows the need to specifically use the larval stage from the infected snails (sporocysts, rediae, or cercariae), to increase the quantity and quality of DNA to be successful in identifying and characterizing the amphistome species found in freshwater snails. There is a need to also develop and apply the use of primers and protocols that do not require sequencing such as the LAMP protocols and PCR-RLFP to discriminate multiple amphistome species of wildlife in IH snails including mixed infections. We recommend future studies such as collecting amphistome specimens from culled wildlife ruminants from the study locations for morphological and molecular identification.

## Figures and Tables

**Table 1:** Description of habitat type and animal/human activity at localities where freshwater snails were collected in the Matebeleland region of Zimbabwe.

Locality	No. of Habitats Surveyed	No. of Habitats with Snails	Habitat Type	Vegetation Cover/Description	Animal/Human Activity
Nyamandlovu	13	3	Waterholes made of concrete (typically less than 40 m^2^)	Sub-merged vegetation in some waterholes but typically some waterholes are clear	Wild ruminants (100%)
West Nicholson	2	2	Dams	A lot of submerged vegetation, trees on the periphery, and decaying organic matter	Wild animals (100%)
Esigodini	1	1	Dam	Submerged vegetation and a lot of trees on the periphery	Wild ruminants (50%), livestock (40%), and human activity (10%)
Ntabazinduna	1	1	Dam	Very little submerged vegetation with no trees on the periphery but a lot of decaying organic matter. More brown algae macrophytes observed.	Wild ruminants (30%), livestock (70%)
Matopos	1	1	Dam	A lot of submerged vegetation with no trees on the periphery	Wild ruminants (80%) and 20% livestock
Inyathi	1	1	River	Few submerged vegetation with trees on the periphery	50% wild ruminants and 50% livestock
Total	19	9			

**Table 2:** Morphological and molecular identities and number of snail species collected in the different localities surveyed in the Matebeleland region of Zimbabwe.

Morphology Identification	Molecular Identification		Number of Snails Collected Per Locality						Total	% Overall Prevalence Per Snail Species
	Species	% Similarity	West Nicholson	Esigodini	Inyathi	Matopos	Nyamandlovu	Ntabazinduna		
*Melanoides tuberculata*	ND	-	58	-	-	-	-	-	58	11.9
*Biomphalaria pfeifferi*	*Biomphalaria pfeifferi*	100	-	13	-	7	-	-	20	4.1
*Bulinus tropicus*	*Bulinus tropicus*	99.78	-	-	3	5	58	99	165	33.9
*Bulinus truncatus*	*Bulinus truncatus*	99.78	-	-	-	-	-	61	61	12.5
*Bulinus globosus*	ND	-	1	5	-	-	-	12	18	8.2
*Physa acuta*	ND	-	-	3	-	-	152	-	155	31.2
*Lymnaea natalensis*	*Lymnaea natalensis*	98.68	-	-	-	6	2	-	8	1.6
*Bellamya* spp.	ND	-	-	-	-	-	-	2	2	0.4
Total			59	21	3	18	212	174	487	
% Prevalence per locality			12.1	4.3	0.6	3.7	43.5	36		

ND = not done.

**Table 3:** Prevalence per locality of amphistome infection in freshwater snails collected from the Matebeleland region of Zimbabwe as detected by PCR.

Gastropod Species	No. of Snails Screened	Snails Positive for Amphistome DNA Per Locality						Total Infected	% Prevalence
		West Nicholson	Esigodini	Inyathi	Matopos	Nyamandlovu	Ntabazinduna		
*Melanoides tuberculata*	58	20	-	-	-	-	-	20	34.5
*Biomphalaria pfeifferi*	20	-	1	-	0	-	-	1	5
*Bulinus tropicus*	165	-	-	0	1	5	14	20	12.1
*Bulinus truncatus*	61	-	-	-	-	-	9	9	14.8
*Bulinus globosus*	18	0	5	-	-	-	3	8	44.4
*Physa acuta*	152	-	-	-	-	0	-	0	0
*Lymnaea natalensis*	12	-	-	-	0	0	-	0	0
*Bellamya* spp.	2	-	-	-	-	-	0	0	0
Total infected		20	6	0	1	5	26	58	
N	487	59	21	3	18	212	174	487	
% Prevalence	-	33.9	28.6	0	5.6	2.4	14.9	-	-

**Table 4:** BLAST percentage similarity of trematode DNA obtained from snails collected in the Matebeleland region of Zimbabwe.

Sample ID	Fragment Size	Species ID Based on Sequence	% Similarity	IH Species (Source)
MO1	290 bp	*Paragonimus kellicotti*	96.10	*Bulinus truncatus*
MB1	290 bp	*Paragonimus kellicotti*	96.42	*Melanoides tuberculata*
MB2	385 bp	*Calicophoron microbothrium*	100	*M. tuberculata*
NT	290 bp	*Paragonimus kellicotti*	96.42	*Bul. truncatus*

## Data Availability

The sequences generated in this study were deposited to GenBank under accession numbers of PP389543-48 and PP392960-63. The alignments used for the phylogenetic analysis are available from the corresponding author on request.
